# A study on the path of governance in health insurance fraud considering moral hazard

**DOI:** 10.3389/fpubh.2023.1199912

**Published:** 2023-09-14

**Authors:** Jusheng Liu, Yuan Wang, Jiali Yu

**Affiliations:** ^1^School of Economics and Management, Shanghai University of Political Science and Law, Shanghai, China; ^2^School of Economics and Management, Shanghai Polytechnic University, Shanghai, China

**Keywords:** health insurance fraud, governance path, moral hazard, evolutionary game, fraud cost

## Abstract

Combating health insurance fraud is of utmost importance to physicians, patients, and health insurers. To delve into the mechanisms of health insurance fraud between doctors and patients, this study employed evolutionary game theory to construct a model that comprehensively considers moral hazard, fraud cost, reward, punishment, bribes from patients, and other factors. Through theoretical analysis and numerical simulation of the model, the study discovered that the evolution of governance behavior in health insurance fraud is closely linked to its initial construction of the payment matrix and the initial selection of parameters for the payment matrix. Additionally, increasing penalties for fraudulent behavior, increasing the cost of fraud for both doctors and patients, and reducing moral hazard for both can effectively drive the final strategy of the system toward a non-fraudulent state. The study aims to provide valuable insights and recommendations to doctors, patients, and medical insurance institutions in establishing a sound governance environment for managing fraud behavior in health insurance.

## 1. Introduction

With the continuous development of China's economy and the improvement of people's living standards, more and more individuals are receiving better healthcare. In recent years, China has introduced a large number of policies to help patients obtain high-quality medical care (1, 2). For instance, in October 2016, the CPC (Communist Party of China) Central Committee and the State Council issued the “Health China 2030” planning outline (http://www.gov.cn/zhengce/2016-10/25/content_5124174.htm), which prioritized health development and incorporated the concept of health promotion into public policy. Additionally, the government aimed to enhance the quality of medical services and promote humanistic care. In October 2017, the 19th National Congress emphasized the importance of providing comprehensive health services to the population and called for improving the national health policy, constructing a population health information service system, standardizing and promoting “Internet + healthcare” services, innovating internet healthcare service models, and advancing intelligent medical and health services (http://www.gov.cn/xinwen/2022-10/25/content_5721685.htm). In July 2019, the General Office of the State Council issued the “Health China Action Organization, Implementation, and Assessment Program” (http://www.gov.cn/zhengce/content/2019-07/15/content_5409499.htm), which required all relevant departments to study major issues related to the Health China Strategy and develop timely policy measures for the Health China Action (http://www.gov.cn/xinwen/2019-07/15/content_5409694.htm). It is evident from these policy measures that the health of the population, as an essential component to promoting a healthy China, has always been present throughout the process of policy formulation in China.

China's healthcare system has experienced significant improvements in recent years thanks to supportive policies and strategies. For instance, the average life expectancy has increased from 74.8 to 78.2 years over a decade (2011–2021), indicating better overall health for the population. Additionally, China has covered over 1.3 billion people with basic medical insurance and almost 1 billion people with basic pension insurance by 2021, making healthcare more accessible and affordable for all.

However, despite the availability of good medical resources, there are still some unethical behaviors in the healthcare system, most notably health insurance fraud. This occurs when patients and physicians engage in fraudulent practices to reduce personal expenses and obtain greater reimbursements from the government, such as by choosing excessive medical care. Unfortunately, this phenomenon is present in every country or region and can take various forms, including fraudulent treatment, over-limit drug use, and doctor-patient collusion.

Regulating and preventing these types of behaviors is crucial as they can have significant negative impacts on both individuals and the government. Therefore, constructing an effective governance system to combat health insurance fraud is of utmost importance. By implementing strict regulations and monitoring systems, healthcare professionals can be held accountable for any fraudulent activities they engage in, ensuring that patients receive appropriate and necessary medical care while minimizing the financial burden on taxpayers. Overall, it is essential to continue improving healthcare systems while also addressing unethical behaviors to ensure that everyone has access to high-quality medical care.

Up to now, scholars have approached the issue of health insurance fraud from various angles, including identifying the characteristics of fraudsters, discussing prevention measures, exploring factors associated with fraud losses, and proposing theoretical models to understand the phenomenon. Pande and Maas (3) found that family practitioners and psychiatrists were the main groups committing health insurance fraud. Stowell et al. (4) highlighted the ongoing threat of healthcare fraud to the US economy and the public and discussed efforts to prevent such fraud. Timofeyev and Jakovljevic (5) investigated factors associated with fraud loss and identified Medicaid, collusion, and fraudsters' age as relevant factors. Stiernstedt and Brooks (6) focused on developing fraud resilience in the private insurance healthcare market. Fei et al. (7) applied the evolutionary game theory to study the formation of health insurance fraud in China and suggested that insured individuals and patients can actively supervise and prevent collusion with medical institutions to reduce the likelihood of fraud. Furthermore, Haruddin et al. (8) considered that the causes of health insurance fraud in hospitals comes from financial motives, internal controls, revenue targets, leadership, and incentive systems. Ribeiro et al. (9) suggested that insurance fraud is an increasing problem with major financial, societal and humanitarian impact. Flynn (10) explored the health care fraud in Australia and believed that on-line claiming platforms is a major threat to the integrity of their insurance system. Meanwhile, Privacy Act hinders health care fraud investigations in Australia. Stelfox and Redelmeier (11) considered that Medical Insurance fraud is an important reason for the waste of medical expenses. In Canada, after the death of some patients, their families use their information still commit medical fraud. These studies collectively contribute to our understanding of health insurance fraud and provide valuable insights for policymakers and practitioners.

Besides, some studies focus on the detection of healthcare fraud. Scholars used different technical approaches such as blockchain, deep learning, and unsupervised multivariate analysis to detect Medicare fraud. Specifically, Tsai et al. (12) proposed a fraud detection framework for Medicare claims using data mining techniques. Shi et al. (13) used a deep learning algorithm to detect fraudulent healthcare claims. Li et al. (14) developed a model based on the deep belief network to detect healthcare fraud. Thaifur et al. (15) proposed a hybrid approach combining deep learning and rule-based methods for detecting healthcare fraud. Additionally, some studies also use blockchain technology to explore the detection of healthcare fraud. For example, Saldamli et al. (16) proposed a blockchain-based fraud detection system for healthcare claims. Kapadiya et al. (17) developed a blockchain-based model for the secure sharing of healthcare data and detecting fraudulent transactions. Moreover, Settipalli and Gangadharan (18) proposed an unsupervised multivariate analysis model for detecting Medicare fraud and demonstrated its effectiveness. Bauder and Khoshgoftaar (19) explored the challenges of imbalanced big data in Medicare fraud detection and provided insights into addressing the class imbalance. Ai et al. (20) reviewed various fraud detection methodologies in healthcare, including rule-based, statistical, machine-learning, and deep-learning approaches, and discussed their advantages and limitations. Overall, these studies provide useful insights and methods for detecting healthcare fraud, including Medicare fraud.

Scholars have conducted relevant research on Medicare fraud from different perspectives, but few have focused on the behavior mechanism and psychological factors involved in Medicare fraud. In fact, moral hazard is an important issue that can influence patients and physicians to carry out Medicare fraud. Researchers have explored moral hazards from various perspectives, including ex-ante moral hazards in health insurance. For instance, Dave and Kaestner (21) concluded that large moral hazard effects of insurance can increase unhealthy behaviors among older individuals. Einav and Finkelstein (22) confirmed that moral hazard exists in health insurance, and without health insurance, individuals consume less healthcare. Dong (23) found that having insurance can encourage and increase unhealthy behavior via moral hazard. Soofi et al. (24) concluded that moral hazard in health insurance can lead to individuals paying for unnecessary care services, hospitals increasing healthcare expenditures, and society losing social welfare. Therefore, the role of moral hazard in health insurance is complex for individuals and society. Although scholars have explored moral hazards in health insurance, there is a need for more research focusing on the behavior mechanism of moral hazard in health insurance fraud. In fact, health insurance fraud is a dynamic and evolutionary process (7). Generally speaking, if government supervision is strict, individuals and hospitals may be deterred from committing health insurance fraud. However, once loopholes in the regulatory or health insurance system exist, driven by moral hazard, individuals may commit health insurance fraud. Thus, a dynamic evolutionary method can better explain the behavior mechanism in health insurance fraud.

At present, evolutionary game theory has proven to be a useful tool for understanding how behavior evolves under conditions of limited rationality. Researchers have applied this theory to a range of phenomena in different fields. For example, Liu et al. (25) used it to explore doctor-patient relationships, while Zhang et al. (26) applied it to the post-pandemic governance of chronic disease diagnosis and treatment systems. In the past, evolutionary game theory has been used to examine the government's safety supervision in the coal mine industry (27), urban public crisis governance (28), and low-carbon technology transfer (29), among other areas. It can be seen that evolutionary game theory provides a useful framework to analyze the behavior and strategy of different objects. The application of evolutionary game theory can help identify the factors that affect individuals' behavior toward health insurance fraud, it also can provide valuable insights into the prevention of the occurrence of health insurance fraud.

It is evident that numerous studies have been conducted on healthcare fraud, yet few scholars have delved into the various types and behaviors of fraudulent practices from a dynamic perspective, with a particular emphasis on their gaming characteristics. Moreover, existing research does not account for factors such as doctor-patient collusion, patient bribery of doctors, and doctor-patient moral hazard in healthcare fraud, which significantly impact the formation of fraudulent schemes. Additionally, previous studies have overlooked the role played by physicians' colleagues in healthcare fraud. To address these gaps, this paper aims to explore the formation mechanism and governance path of medical fraud from moral hazard, focusing on two major game subjects: doctors and patients.

Our study has the following innovations: firstly, we take into account the moral hazard of patients in health insurance fraud, which is often overlooked in previous studies, providing a more accurate representation of fraudulent insurance practices. Secondly, we explore the behavior of health insurance fraud from a dynamic perspective, unlike traditional research that investigates it from a static perspective. Thirdly, we recognize that physicians are also significantly responsible for fraudulent insurance practices, which is often overlooked in traditional research that tends to blame patients for fraud. Finally, this paper analyzes different types of healthcare fraud and considers bribery by patients and collusion between doctors and patients.

**Figure 1:**
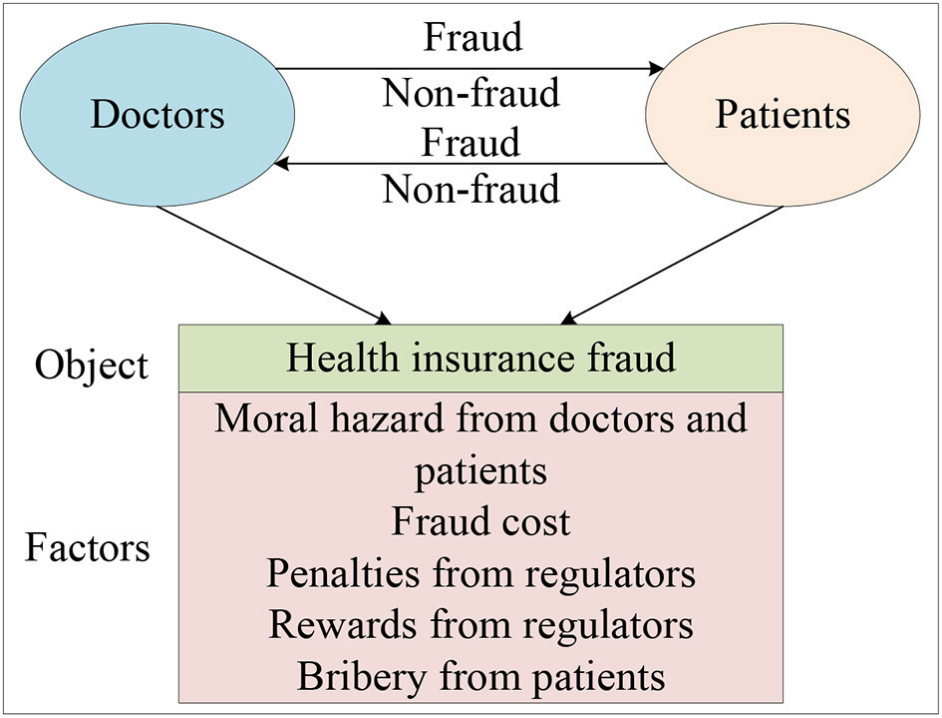
Research framework.

Distinct from the empirical research method, this paper used the theoretical research method named evolutionary game theory to explore the evolution patterns of governance paths in the behavior of health insurance fraud. Unlike traditional game theory, evolutionary game theory does not require participants to be perfectly rational, nor does it require the condition of perfect information. In terms of methodology, it differs from game theory in that it focuses on static equilibrium and comparative static equilibrium, emphasizing a dynamic equilibrium. Generally speaking, the evolutionary game model has the following characteristics: firstly, taking the participant group as the object of study, it analyzes the dynamic evolutionary process and explains why and how the group has reached and how it has reached this current state; Secondly, the evolution of the group has both selection and mutation processes; Thirdly, behavior selected down by the group has a certain degree of inertia. By applying evolutionary game theory to explore the evolution patterns of governance paths in the behavior of health insurance fraud, our findings indicate that the evolution of the governance behavior of the doctors and patients is closely linked to the payment matrix they initially constructed and the initial parameter selection of the payment matrix. Our study suggests that increasing penalties for fraud behavior by doctors and patients, increasing the cost of fraud from doctors and patients, and reducing the moral hazard of doctors and patients can effectively promote the system to stabilize in the (non-fraud, non-fraud) state. Ultimately, our goal is to provide references and suggestions for medical insurance institutions and individuals to combat health insurance fraud. The research framework is presented in Figure 1.

The rest of this paper is organized as follows. Basic assumptions are proposed and a model is built in Section 2. In Section 3, we conducted a numerical simulation analysis, demonstrating the consistency between the numerical simulation results and theoretical derivation results. Finally, the entire article is summarized and suggestions are provided in Section 4.

## 2. Basic assumptions and model construction

**Figure 2:**
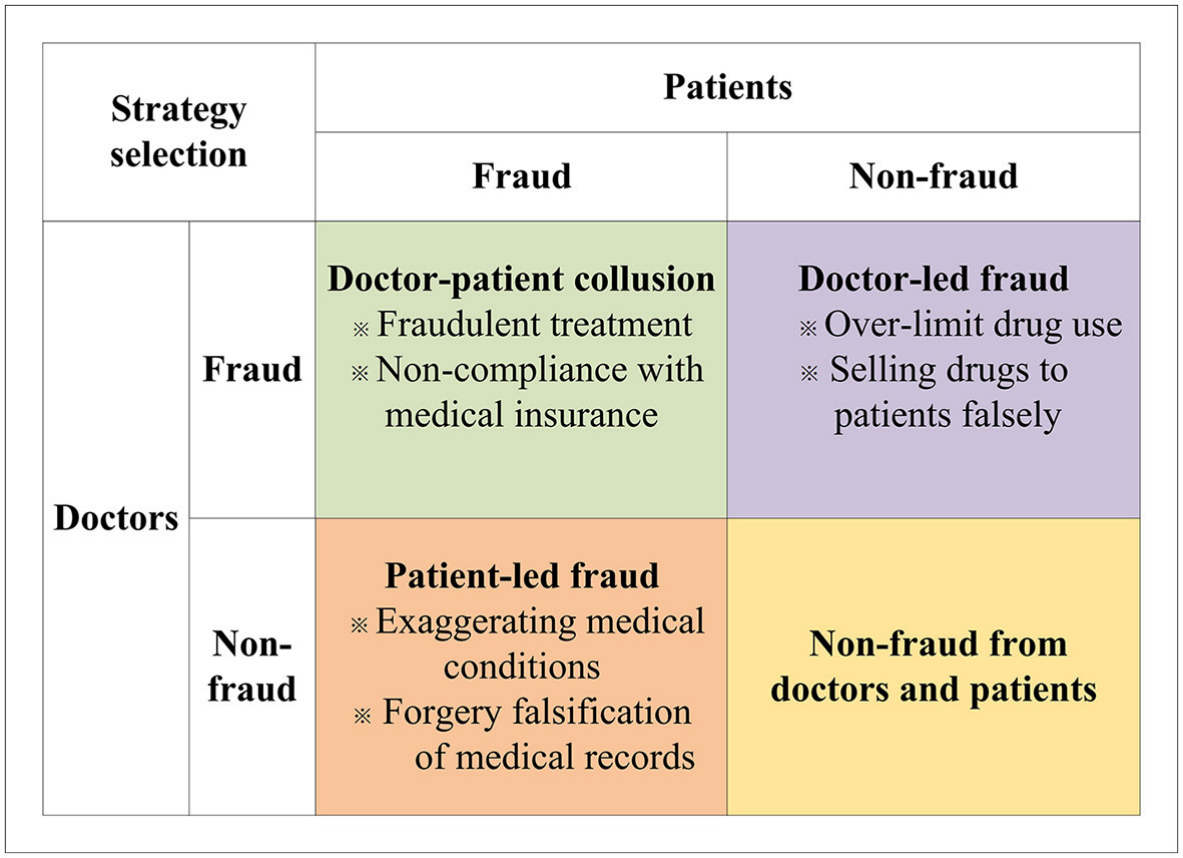
Doctor-patient health insurance fraud types.

Health insurance fraud is a pervasive problem that has wide-ranging negative effects on society, including loss of social benefits, erosion of trust in the integrity of the system, and the creation of a fertile ground for criminal activity. Therefore, how establishing an honest and transparent health insurance system between doctors and patients is important. In this study, we investigate the behavior of patients and doctors in the context of health insurance fraud by taking into account factors such as moral hazard from doctors and patients, penalties and rewards from insuranceinstitutions, bribey from patients, and other factors. To display the behavior mechanism and clarify the problem, some fraud behaviors between doctors and patients are discussed in Figure 2.

As shown in Figure 2, in real life, according to the behavior of doctors and patients, there are three types of health insurance fraud, patient-led, doctor-led, and doctor-patient collusion. When the patients choose the fraud behavior, but the doctors choose the non-fraud behavior, patient-led fraud will occur. Such fraud includes exaggerating medical conditions and forgery and falsification of medical records. When the doctors choose fraudulent behavior, but the patients choose non-fraud behavior, doctor-led fraud will ensue. In such cases, the doctors will over-prescribe drugs and induce patients to overpay for medicine. Further, when the doctors and patients both choose fraudulent behavior, doctor-patient collusion will occur. In this mode, the doctors and patients will build consensus to work together on healthcare fraud. For example, doctors and patients will fail to comply with medical insurance regulations, taking advantage of false facts to arbitrage medical insurance fees. The doctors may do fraudulent treatment with patients and use fake prescriptions to obtain more health insurance. At this point, doctors and patients will gain the most from their perspective compared to patient-led and physician-led healthcare fraud.

To further explore the mechanism of medical insurance fraud between doctors and patients, the following assumptions are made in this study:

**Assumption 1**: In a health insurance fraud system, patients and doctors are the primary participants and the medical insurance institutions play the role of regulators. The doctors have two strategic options for dealing with health insurance fraud: fraud and non-fraud, with a probability of *x* and 1 − *x*, respectively. Similarly, patients also have two strategic options: fraud and non-fraud, with a probability of *y* and 1 − *y*, respectively. When the doctors choose non-fraud and patients choose non-fraud, the doctors receive a payoff of *K*_0_ and the patients receive a payoff of *N*_0_.

**Assumption 2**: When physicians and patients commit Medicare fraud, they will pay a cost of fraud. For example, doctors may be penalized if they perform fraudulent treatment and induce patients to overpay for medicine. Similarly, if patients overuse medical resources, for example, if they remain hospitalized and do not pay for medical treatment even though their condition has been cured, they will pay certain costs, such as reputational and time costs. Generally, it is assumed that the doctors' cost of fraud is *C*_1_, while patients' cost of fraud is *C*_2_. Moreover, to encourage doctors and patients to participate in the health insurance system honestly, the medical insurance institutions will offer them a certain reward denoted as *R*_1_ and *R*_2_ for doctors and patients. Similarly, when doctors and patients commit health care fraud, they are also penalized by the insurance agency, with a penalty of *P*_1_ for the doctors and *P*_2_ for the patients.

**Assumption 3**: In the real world, if doctors commit medical fraud, they will receive certain benefits *K*_1_, and *K*_1_ > *K*_0_. Similarly, if the patients commit medical fraud, they will receive certain benefits *N*_1_, and *N*_1_ > *K*_0_. Besides, moral hazard is an important factor that influences the behavior of doctors and patients under information asymmetry. When the patients choose the non-fraud strategy, the doctors choose fraud. This moment, the doctors will commit medical fraud by prescribing medicines under moral hazard. For the same reason, patients also have moral hazard. For example, patients may exaggerate their condition and lie to doctors as well as more about their treatment without overpaying for medical care under the moral hazard. To facilitate the moral hazard for physicians and patients, we assume that the moral hazard coefficient of doctors is denoted as *f* and the moral hazard coefficient of patients is denoted as *g*. If the doctors select fraud, but patients select the non-fraud. Under the information asymmetry, the doctors will falsely prescribe drugs to make patients pay more for care and obtain additional benefit *fN*_0_ from patients. Similarly, if the doctors select the non-fraud, but patients select the fraud, the patients will obtain additional benefit *gK*_0_ from doctors.

**Assumption 4**: In the Medicare fraud process, the doctors and patients will be exposed to certain risks. It is assumed that the risk coefficients of doctors and patients are β_1_ and β_2_. Risk often has a positive correlation with returns, thus, if a doctor engages in medical fraud, the risk he will face is β_1_*K*_1_, similarly, if a patient engages in medical fraud, the risk he will face is β_2_*N*_1_.

**Assumption 5**: In medical fraud, it is different from single patient-led fraud and doctor-led fraud, patients can bribe doctors, prompting both doctors and patients to form collusion and engage in medical fraud together. We assume that patients' bribery of doctors is *H*. Besides, when doctors and patients form collusion, they will obtain extra benefits from health insurance instructions, compared with patient-led fraud and doctor-led fraud. At this moment, it is assumed that the extra benefit is *M*. Furthermore, we assume the benefit coefficient for doctors is α and the benefit coefficient for patients is 1−*α*. To provide a clear understanding of each parameter in the above assumptions, Table 1 presents a comprehensive description of these parameters.

**Table 1:** Notations and interpretations.

**Notations**	**Interpretations**
*x*	Probability of the doctors choosing fraud strategy
*y*	Probability of the patients choosing fraud strategy
*K*_0_	Doctors' payoff when they choose non-fraud strategy
*K*_1_	Doctors' payoff when they choose fraud strategy (*K*_0_<*K*_1_)
*N*_0_	Patients' payoff when they choose non-fraud strategy (*N*_0_<*N*_1_)
*N*_1_	Patients' payoff when they choose fraud strategy
*C*_1_	Doctors' fraud cost
*C*_2_	Patients' fraud cost
*f*	Moral hazard coefficient of doctors (0 < *f* < 1)
*g*	Moral hazard coefficient of patients (0 < *g* < 1)
*P*_1_	Punishment of the insurance instructions toward the doctors when they choose fraud strategy
*P*_2_	Punishment of the insurance instructions toward the patients when they choose fraud strategy
*R*_1_	Reward of the insurance instructions toward the doctors when they choose non-fraud strategy
*R*_2_	Reward of the insurance instructions toward the patients when they choose non-fraud strategy
β_1_	Risk coefficient of doctors when they choose fraud strategy (0 < β_1_ < 1)
β_2_	Risk coefficient of patients when they choose fraud strategy (0 < β_2_ < 1)
*H*	Bribery given by patients to doctors
*M*	Extra benefit from insurance instructions when doctors and patients form a collusion
α	Benefit coefficient for doctors when doctors and patients form a collusion (0 < α < 1)

### 2.1. Construction of payoff matrix for evolutionary game

Table 2 presents the payoff matrix between the government and individuals, based on the assumptions described above.

**Table 2:** Payment matrix of patient and doctor.

**Strategy selection**		**Patient (** ***j*** **)**	
		**Frand(** *y* **)**	**Non-fraud (**1−*y***)**
Doctor(*i*)	Fraud (*x*)	(−*C*_1_+*H*+α*M*+*K*_1_−β_1_*K*_1_−*P*_1_),	(−*C*_1_+*fN*_0_+*K*_1_−*P*_1_−β_1_*K*_1_),
		(−*C*_2_+*H*+(1−α)*M*+*N*_1_−β_2_*N*_1_−*P*_2_)	(*N*_0_+*R*_2_−*fN*_0_)
	Non-fraud (1−*x*)	(*K*_0_+*R*_1_−*gK*_0_),	(*K*_0_),
		(−*C*_2_+*gK*_0_−*P*_2_−β_2_*N*_1_+*N*_1_)	(*N*_0_)

At the time *t*, the benefit that the doctors receive from choosing a fraud strategy is represented by *U*_11_:


(1) $$\begin{array}{l} U_{11}=y(-C_{1}+H+\alpha M+K_{1}-\beta_{1}K_{1}-P_{1})+(1-y)\\ \;(-C_{1}+fN_{0}+K_{1}-P_{1}-\beta_{1}K_{1}) \end{array}$$

The benefit that the doctors receive from choosing a non-fraud strategy is represented by *U*_12_:


(2) $$\begin{array}{l} U_{12}=y\left(K_{0}+R_{1}-gK_{0}\right)+\left(1-y\right)K_{0} \end{array}$$

The average benefit to doctors of choosing both fraud and non-fraud strategies is represented by $\bar{U_{1}}$:


(3) $$\begin{array}{l} \bar{U_{1}}=xU_{11}+\left(1-x\right)U_{12} \end{array}$$

The benefit that patients receive from choosing the fraud strategy in health insurance is represented by *U*_21_.


(4) $$\begin{array}{l} U_{21}=x(-C_{2}-H+(1-\alpha )M+N_{1}-\beta_{2}N_{1}-P_{2})+(1-x)\\ \;(-C_{2}+gK_{0}-P_{2}-\beta_{2}N_{1}+N_{1}) \end{array}$$

The benefit that patients receive from choosing the non-fraud strategy in health insurance is denoted as *U*_22_:


(5) $$\begin{array}{l} U_{22}=x\left(N_{0}+R_{2}-fN_{0}\right)+\left(1-x\right)N_{0} \end{array}$$

The average benefit to patients of choosing both fraud and non-fraud strategies in health insurance is represented by $\bar{U_{2}}:$


(6) $$\begin{array}{l} \bar{U_{2}}=yU_{21}+\left(1-y\right)U_{22} \end{array}$$

The replication dynamic equation is a type of differential equation that is effective in modeling and replicating the evolutionary characteristics of a previous strategy. It guides the population toward a direction that is conducive to its development (30–32). This equation is commonly used to assess the dynamic trend of strategy evolution within a population and is typically expressed as follows:


(7) $$\begin{array}{l} F\left(x\right)=\frac{dx}{dt}=x\left(U_{n}-Ū\right),x\in \left[0,1\right] \end{array}$$

Where *x* represents the proportion of individuals in the population using a certain strategy *n*; *U*_*n*_ represents the expected payoff of the player using strategy *n*; and *Ū* represents the average expected payoff of the player's overall strategies.

According to the replication dynamic equation, at time *t*, the replicator dynamic equations for the doctor and patient are:


(8) $$\{\begin{array}{l} F(x,y)=\frac{dx}{dt}=x(1-x)[-C_{1}+fN_{0}+K_{1}-P_{1}-\beta_{1}K_{1}-K_{0}+\\ y(H+\alpha M-R_{1}+gK_{0}-fN_{0})]\\ G(x,y)=\frac{dy}{dt}=y(1-y)[-C_{2}+gK_{0}+N_{1}-P_{2}-\beta_{2}N_{1}-N_{0}+\\ y(-H+(1-\alpha )M-R_{2}-gK_{0}+fN_{0})] \end{array}$$

Let *F*(*x, y*) = 0 and *G*(*x, y*) = 0, we can obtain five equilibrium points for evolutionary game systems, namely *E*(0, 0), *S*(0, 1), *N*(1, 0), *K*(1, 1), and *P*(x^*^, y^*^), where


(9) $$\begin{array}{l} x^{*}=\frac{C_{2}-gK_{0}-N_{1}+P_{2}+\beta_{2}N_{1}+N_{0}}{-H+(1-\alpha )M-R_{2}-gK_{0}+fN_{0}},\\ y^{*}=\frac{C_{1}-fN_{0}-K_{1}+P_{1}+\beta_{1}K_{1}+K_{0}}{H+\alpha M-R_{1}+gK_{0}-fN_{0}} \end{array}$$

To explore the evolution of the different paths between governments and individuals in the system of health insurance fraud, we can analyze the Jacobian matrix for an evolutionary game under different conditions and cases. The Jacobian matrix *J* can be obtained by differentiating the replicator dynamic equation concerning *x* and *y*. Specifically, we have:


$$J=\left[\begin{array}{cc} \frac{\partial F(x,y)}{\partial x} & \frac{\partial F(x,y)}{\partial y}\\ \frac{\partial G(x,y)}{\partial x} & \frac{\partial G(x,y)}{\partial y} \end{array}\right]$$

(10) $$\begin{array}{l} \frac{\partial F(x,y)}{\partial x}=(1-2x)[-C_{1}+fN_{0}+K_{1}-P_{1}-\beta_{1}K_{1}-K_{0}+y\\ \;(H+\alpha M-R_{1}+gK_{0}-fN_{0})] \end{array}$$

(11) $$\begin{array}{lll} \frac{\partial F\left(x,y\right)}{\partial y} & = & x\left(1-x\right)\left(H+\alpha M-R_{1}+gK_{0}-fN_{0}\right) \end{array}$$

(12) $$\begin{array}{lll} \frac{\partial G\left(x,y\right)}{\partial x} & = & y\left(1-y\right)\left(-H+\left(1-\alpha \right)M-R_{2}+fN_{0}-gK_{0}\right) \end{array}$$

(13) $$\begin{array}{l} \frac{\partial G(x,y)}{\partial y}=(1-2y)[-C_{2}+gK_{0}+N_{1}-P_{2}-\beta_{2}N_{1}-N_{0}\\ \;+x(-H+(1-\alpha )M-R_{2}-gK_{0}+fN_{0})] \end{array}$$

Using the Jacobian matrix, we can calculate the determinant and trace values at each equilibrium point *E*(0, 0), *S*(0, 1), *N*(1, 0), *K*(1, 1), and *P*(x^*^, y^*^). When the equilibrium point is *E*(0, 0), the Jacobian matrix of *E*(0, 0) is


$$\begin{array}{l} \;J_{E(0,0)}=\\ \left[\begin{array}{cc} -C_{1}+fN_{0}+K_{1}-P_{1}-\beta_{1}K_{1}-K_{0} & 0\\ 0 & -C_{2}+gK_{0}+N_{1}-P_{2}-\beta_{2}N_{1}-N_{0} \end{array}\right]. \end{array}$$

When the equilibrium point is *S*(0, 1), the Jacobian matrix of *S*(0, 1) is


$$\begin{array}{l} \;J_{S(0,1)}=\\ \left[\begin{array}{cc} \begin{array}{l} (-C_{1}+fN_{0}+K_{1}-P_{1}-\beta_{1}K_{1}-K_{0}+H+\\ \;\alpha M-R_{1}+gK_{0}-fN_{0}) \end{array} & 0\\ 0 & -(-C_{2}+gK_{0}+N_{1}-P_{2}-\beta_{2}N_{1}-N_{0}) \end{array}\right]. \end{array}$$

When the equilibrium point is *N*(1, 0), the Jacobian matrix of *N*(1, 0) is


$$\begin{array}{l} \;J_{N(1,0)}=\\ \left[\begin{array}{cc} -(-C_{1}+fN_{0}+K_{1}-P_{1}-\beta_{1}K_{1}-K_{0}) & 0\\ 0 & \begin{array}{l} {}[-C_{2}+gK_{0}+N_{1}-P_{2}-\beta_{2}N_{1}-N_{0}\\ -H+(1-\alpha )M-R_{2}+fN_{0}-gK_{0}] \end{array} \end{array}\right]. \end{array}$$

When the equilibrium point is *K*(1, 1), the Jacobian matrix of *K*(1, 1) is


$$\begin{array}{l} \;J_{K(1,1)}=\\ \left[\begin{array}{cc} \begin{array}{l} -(-C_{1}+fN_{0}+K_{1}-P_{1}-\beta_{1}K_{1}-K_{0}\\ \;+H+\alpha M-R_{1}-fN_{0}+gK_{0}) \end{array} & 0\\ 0 & \begin{array}{l} -[-C_{2}+gK_{0}+N_{1}-P_{2}-\beta_{2}N_{1}-N_{0}\\ \;-H+(1-\alpha )M-R_{2}+fN_{0}-gK_{0}] \end{array} \end{array}\right]. \end{array}$$

When the equilibrium point is *P*(x^*^, y^*^), the Jacobian matrix of *P*(x^*^, y^*^) is


$$\begin{array}{l} \;J_{P(x^{*},y^{*})}=\\ \;\left[\;\begin{array}{cc} 0 & \;x^{*}(1-x^{*})(H+\alpha M-R_{1}-fN_{0}+gK_{0})\\ y^{*}(1-y^{*})[H+(1-\alpha )M-R_{2}+fN_{0}-gK_{0}] & \;0 \end{array}\;\right]. \end{array}$$

### 2.2. Analysis of payoff matrix for evolutionary game

To explore the evolutionary status of the different paths between doctors and patients in the system of health insurance fraud, this study analyzes the matrix for an evolutionary game under different conditions and cases. Three cases can be obtained in this research based on the different values of x^*^ and y^*^.

**Case (1)** when *C*_2_ − *gK*_0_ + *P*_2_ + *β*_2_*N*_1_ − *N*_1_ + *N*_0_ > − *H* + (1 − *α*)*M* − *R*_2_ + *fN*_0_ − *gK*_0_ > 0 and *C*_1_ − *fN*_0_ + *P*_1_ + *β*_1_*K*_1_ − *K*_1_ + *K*_0_ > *H* + *αM* − *R*_1_ − *fN*_0_ + *gK*_0_ > 0, we have x^*^ > 1 and y^*^ > 1. Four equilibrium points exist in the system, namely *E*(0, 0), *S*(0, 1), *N*(1, 0), and *K*(1, 1). To determine the stability of these points, the Jacobian matrix's determinant and trace are calculated at each point using the system stability criterion. A point is stable if its determinant is positive and the trace is negative, while it is unstable if the determinant is positive and the trace is positive. If the determinant is negative and the trace is uncertain or if the determinant is positive and the trace is zero, the point is a saddle point. The stability of the system can be assessed based on the determinant and trace symbols, as illustrated in Table 3.

**Table 3:** Sign of the determinant and trace of the Jacobian matrix at each equilibrium point in case (1).

**Equilibrium point**	**Sign of determinant**	**Sign of trace**	**Stability**
*E*(0, 0)	+	−	ESS
*S*(0, 1)	−	Uncertain	Saddle point
*N*(1, 0)	−	Uncertain	Saddle point
*K*(1, 1)	+	+	Unstable

**Figure 3:**
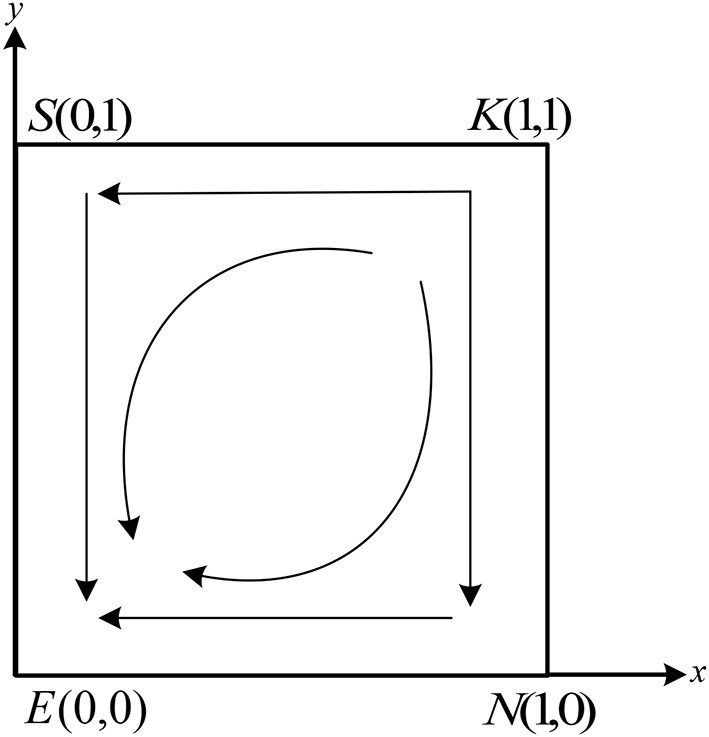
Systematic evolutionary phase diagram in case (1).

Table 3 shows that the system has two saddle points, *S*(0, 1) and *N*(1, 0), one stable point, *E*(0, 0), and one unstable point, *K*(1, 1). Based on the conditions in case (1), it can be deduced that − *C*_2_ − *H* + (1 − α)*M* + *N*_1_ − *β*_2_*N*_1_ − *P*_2_ < *N*_0_ + *R*_2_ − *fN*_0_, − *C*_2_ + *gK*_0_ − *P*_2_ − *β*_2_*N*_1_ + *N*_1_ < *N*_0_. This implies that the benefit of the non-fraud strategy is larger than that of the fraud strategy. Thus, whatever strategy the doctors choose, the patients choose the non-fraud strategy. Similarly, according to the conditions in case (1), we have − *C*_1_ + *H* + α*M* + *K*_1_ − *β*_1_*K*_1_ − *P*_1_ < *K*_0_ + *R*_1_ − *gK*_0_, − *C*_1_ + *fN*_0_ + *K*_1_ − *P*_1_ − *β*_1_*K*_1_ < *K*_0_. According to the payoff in Table 2, the benefit of non-fraud is larger than fraud. Therefore, whatever strategy the patients choose, the doctors will select the strategy of non-fraud. The final evolutionarily stable strategy (ESS) stabilizes at point *E*(0, 0). The phase portrait of the system under these conditions is shown in Figure 3.

As shown in Figure 3, during the evolution process, the unstable point *K*(1, 1) in the system will move toward the stable point *E*(0, 0) along the path *KSE* and path *KNE*, and eventually stabilize at point *E*(0, 0).

**Case (2)** When *C*_2_ − *gK*_0_ + *P*_2_ + *β*_2_*N*_1_ − *N*_1_ + *N*_0_ < 0, − *H* + (1 − *α*)*M* − *R*_2_ + *fN*_0_ − *gK*_0_ > 0 and *C*_1_ − *fN*_0_ + *P*_1_ + *β*_1_*K*_1_ − *K*_1_ + *K*_0_ < 0, *H* + *αM* − *R*_1_ − *fN*_0_ + *gK*_0_ > 0, we have x^*^ < 0 and 0 < y^*^ < 0. At this moment, there are four equilibrium points in the system, which are *E*(0, 0), *S*(0, 1), *N*(1, 0), *andK*(1, 1). The signs of the determinant and trace of the Jacobian matrix at each equilibrium point are shown in Table 4.

**Table 4:** Sign of the determinant and trace of the Jacobian matrix at each equilibrium point in case (2).

**Equilibrium point**	**Sign of determinant**	**Sign of trace**	**Stability**
*E*(0, 0)	+	+	Unstable
*S*(0, 1)	−	Uncertain	Saddle point
*N*(1, 0)	−	Uncertain	Saddle point
*K*(1, 1)	+	−	ESS

**Figure 4:**
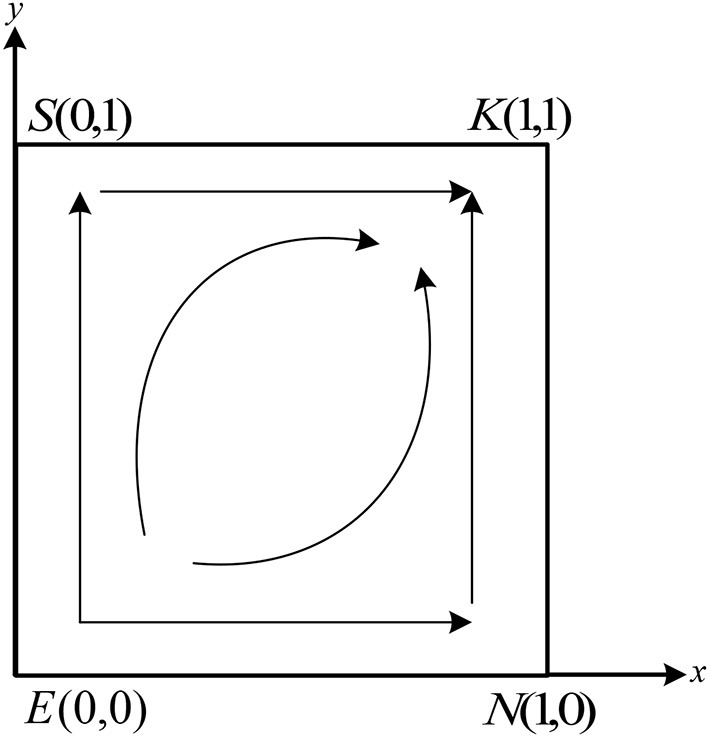
Simulation phase diagram of the evolution of the system for case (2).

As shown in Table 4, there are one stable point, *K*(1, 1), one unstable point *E*(0, 0), and two saddle points *S*(0, 1) and *N*(1, 0) in the system. Therefore, there is one stable strategy in the system. According to the conditions in case (2), it can be further deduced that −*C*_2_ − *H* + (1 − *α*)*M* + *N*_1_ − *β*_2_*N*_1_ − *P*_2_ > *N*_0_ + *R*_2_ − *fN*_0_, − *C*_2_ + *gK*_0_ − *P*_2_ − *β*_2_*N*_1_ + *N*_1_ > *N*_0_. At this moment, whatever strategy the doctors choose, the patients choose the fraud strategy. Similarly, according to the conditions in case (1), we can get − *C*_1_ + *H* + *αM* + *K*_1_ − *β*_1_*K*_1_ − *P*_1_ > *K*_0_ + *R*_1_ − *gK*_0_, − *C*_1_ + *fN*_0_ + *K*_1_ − *P*_1_ − *β*_1_*K*_1_ > *K*_0_. It means whatever strategy the patients choose, the doctors will select the strategy of fraud. From the above results, it can be seen that The final evolutionarily stable strategy (ESS) stabilizes at point *K*(1, 1). The evolution phase diagram of the system is shown in Figure 4.

As shown in Figure 4, the trajectories of the system are moving from *E*(0, 0) to the *K*(1, 1) along the path *ESK* and path *ENK*, and eventually stabilize at point *K*(1, 1).

**Case (3)** when 0 < *C*_2_ − *gK*_0_ + *P*_2_ + *β*_2_*N*_1_ − *N*_1_ + *N*_0_ < − *H* + (1 − *α*)*M* − *R*_2_ + *fN*_0_ − *gK*_0_ and 0 < *C*_1_ − *fN*_0_ + *P*_1_ + *β*_1_*K*_1_ − *K*_1_ + *K*_0_ < *H* + *αM* − *R*_1_ − *fN*_0_ + *gK*_0_, we have 0 < x^*^ < 1 and 0 < y^*^ < 1. At this moment, there are five equilibrium points in the system, which are *E*(0, 0), *S*(0, 1), *N*(1, 0), *K*(1, 1), and *P*(x^*^, y^*^). The signs of the determinant and trace of the Jacobian matrix at each equilibrium point are shown in Table 5.

**Table 5:** Sign of the determinant and trace of the Jacobian matrix at each equilibrium point in case (2).

**Equilibrium point**	**Sign of determinant**	**Sign of trace**	**Stability**
*E*(0, 0)	+	−	ESS
*S*(0, 1)	+	+	Unstable
*N*(1, 0)	+	+	Unstable
*K*(1, 1)	+	−	ESS
*P*(x^*^, y^*^)	−	0	Saddle point

**Figure 5:**
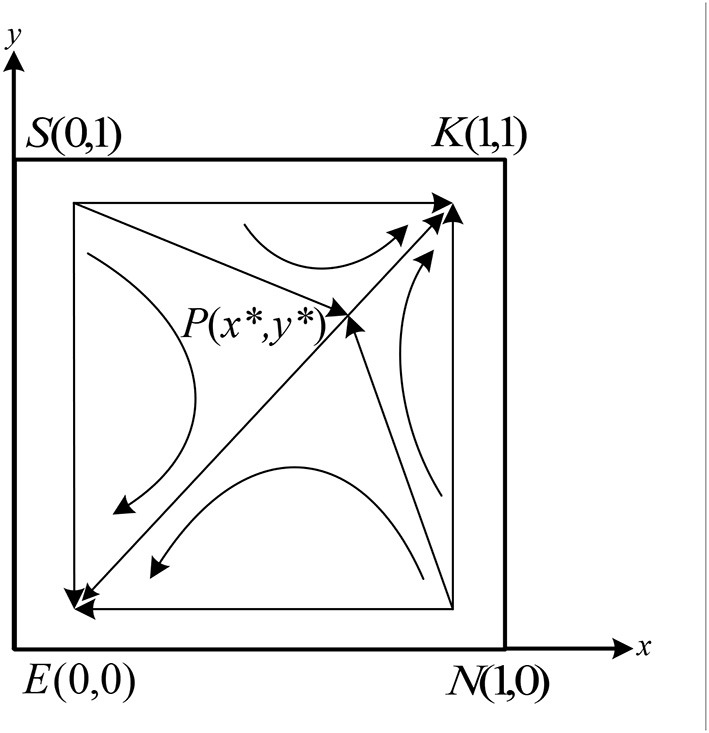
Simulation phase diagram of the evolution of the system for case (3).

As shown in Table 5, there are two stable points *E*(0, 0) and *K*(1, 1), two unstable points *S*(0, 1) and *N*(1, 0), and one saddle point *P*(x^*^, y^*^) in the system. According to the conditions in case (3), we can get −*C*_2_ − *H* + (1 − *α*)*M* + *N*_1_ − *β*_2_*N*_1_ − *P*_2_ > *N*_0_ + *R*_2_ − *fN*_0_, − *C*_2_ + *gK*_0_ − *P*_2_ − *β*_2_*N*_1_ + *N*_1_ < *N*_0_. At this moment, if the doctors choose the fraud strategy, the patients will choose the fraud strategy. If the doctors choose the non-fraud strategy, the patients will also choose the non-fraud strategy. Similarly, according to the conditions in case (3), it can be found that The doctor's strategy will also change according to the patient's strategy. The evolution phase diagram of the system in case(3) is shown in Figure 5.

As shown in Figure 5, the final stabilization strategy of the system will be determined by the area of the quadrilateral enclosed by the *P*(x^*^, y^*^). If 0 < x^*^ < 1/2 and 0 < y^*^ < 1/2, the area of the quadrilateral *S*_*PSEN*_ is smaller than the *S*_*PSKN*_, the final strategy of the system will be stabilized at the point *K*(1, 1). Similarly, if 1/2 < *x* < 1 and 1/2 < *y* < 1, the final strategy of the system will be stabilized at the point *E*(0, 0). Besides, if *x* = 1/2 and *y* = 1/2, the final strategy of the system may be stabilized at the point *K*(1, 1) or at the point *E*(0, 0).

## 3. Numerical simulation analysis

Numerical simulations were conducted to verify the rationality of the model presented in this study. The related parameters were selected based on the assumptions and conditions in case (3). For case (3), the selected initial parameters were *C*_1_ = 3, *C*_2_ = 2, *N*_0_ = 2, *N*_1_ = 5, *K*_0_ = 2, *K*_1_ = 8, *f* = *l* = 0.8, *g* = 0.6, α = 0.5, β_1_ = 0.5, β_2_ = 0.5, *P*_1_ = 5, *P*_2_ = 3, *R*_1_ = 5, *R*_2_ = 3, *H* = 2, and *M* = 25, which satisfy the conditions in case (3). To explore the relevant factors affecting the evolution of the system, factors such as fraud cost, penalty, reward, moral hazard, and bribery were selected for simulation. In the simulation results, the horizontal axis represents the step size of the simulation and the vertical axis represents the proportion of doctors and patients choosing the fraud strategy.

### 3.1. Impact of fraud costs from patients and doctors

**Figure 6:**
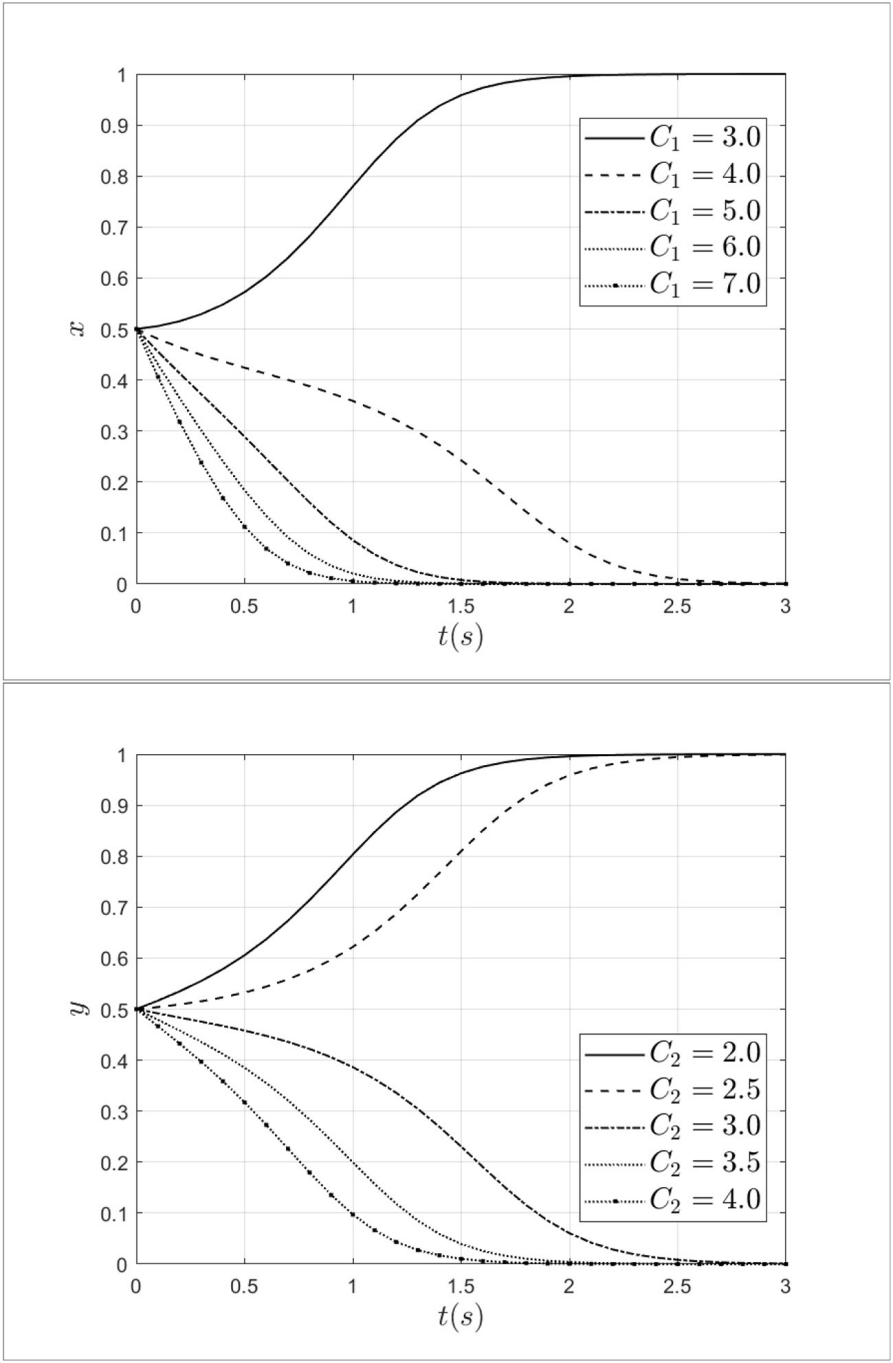
The impact of fraud costs from patients and doctors on the process of system evolution.

Keeping other parameters constant, the fraud cost of the doctors *C*_1_ is taken 3.0, 4.0, 5.0, 6.0, 7.0 and the fraud cost of the patients *C*_2_ is taken 2.0, 2.5, 3.0,3.5, 4.0 separately. The impact of fraud costs from patients and doctors on the process of system evolution is shown in Figure 6.

As shown in Figure 6, it can be seen that when the fraud cost of doctor *C*_1_ increases from 3.0 to 7.0, the doctor's strategy will shift from fraud to non-fraud, and the rate of evolution is gradually accelerating. Thus, from the above results, it can be seen that the increased cost of fraud will discourage fraudulent behavior by doctors. Similarly, when the fraud cost of patients *C*_2_ increases from 2.0 to 4.0, the patient's strategy will shift from fraud to non-fraud, and the rate of evolution is gradually accelerating too. The results show that the fraud cost of patients has a negative effect on the evolution of the system. Therefore, by increasing the fraud cost of doctors and patients, the behavior of medical fraud will be reduced.

### 3.2. Impact of moral hazard from patients and doctors

**Figure 7:**
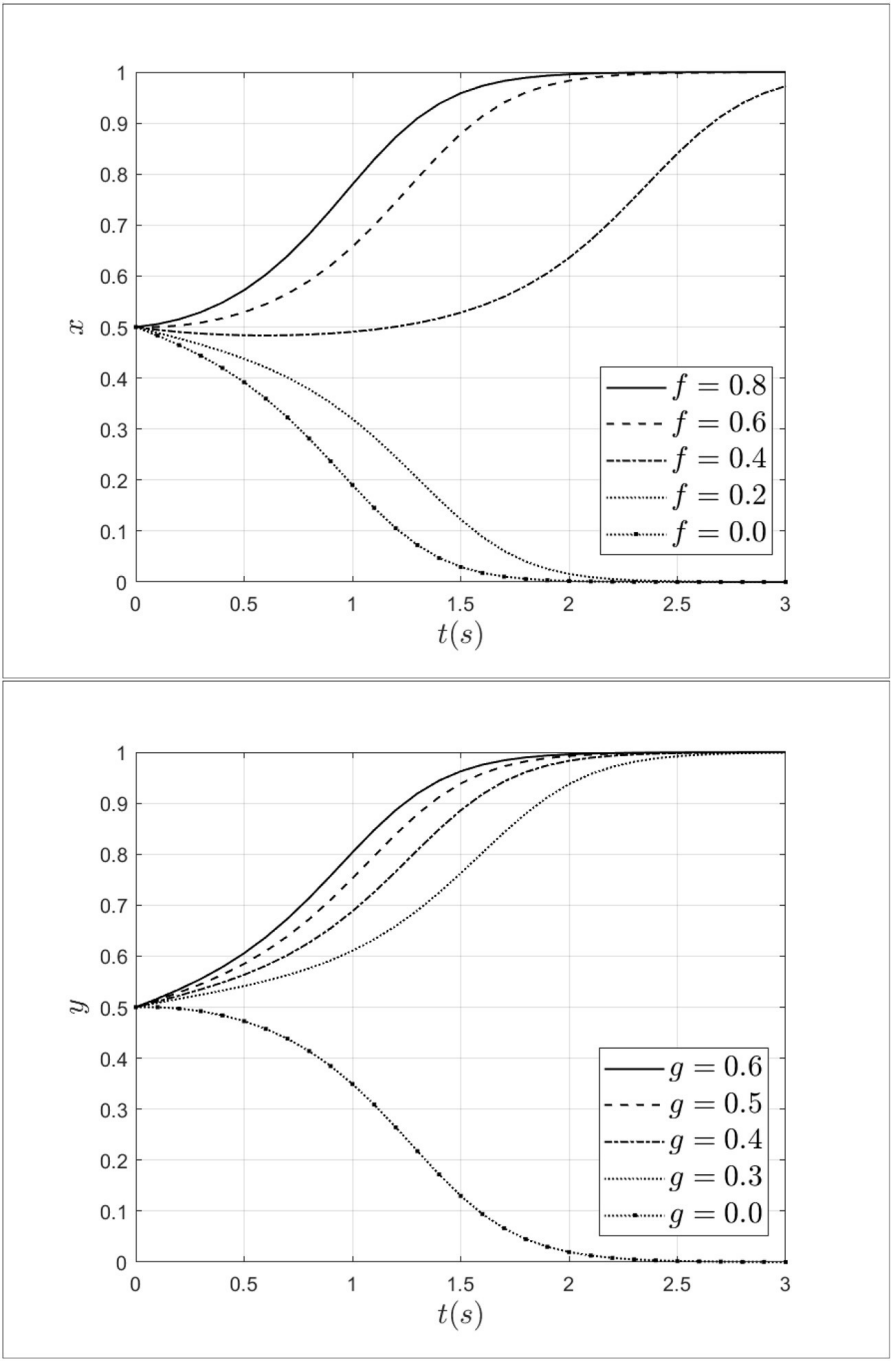
Impact of moral hazard from patients and doctors on the process of system evolution.

As an important factor, moral hazard has a significant impact on medical fraud by both doctors and patients. To explore the impact of moral hazard on the evolution of the system, the moral hazard of the doctor's *f* is taken 0.8, 0.6, 0.4, 0.2, 0.0, and the moral hazard of the patient's *g* is taken 0.6, 0.5, 0.4, 0.3, 0.0. The impact of moral hazard from patients and doctors on the process of system evolution is shown in Figure 7.

As shown in Figure 7, when the moral hazard of a doctor decreases from 0.8 to 0.0, the doctor's strategy will move from fraud strategy to non-fraud strategy and the rate of moving is gradually accelerating. This indicates that the moral hazard of the doctors can influence doctors' behavior effectively. Similarly, when the moral hazard of the patient decreases from 0.6 to 0.0, the strategy of patients will evolve toward a non-fraud strategy. This result indicates that when the moral hazard of patients decreases, the probability of patients choosing fraudulent strategies will gradually decrease. Thus, reducing moral hazard can effectively drive a shift in strategy choice from fraud to non-fraud strategy for both doctors and patients.

### 3.3. Impact of penalty for patients and doctors

**Figure 8:**
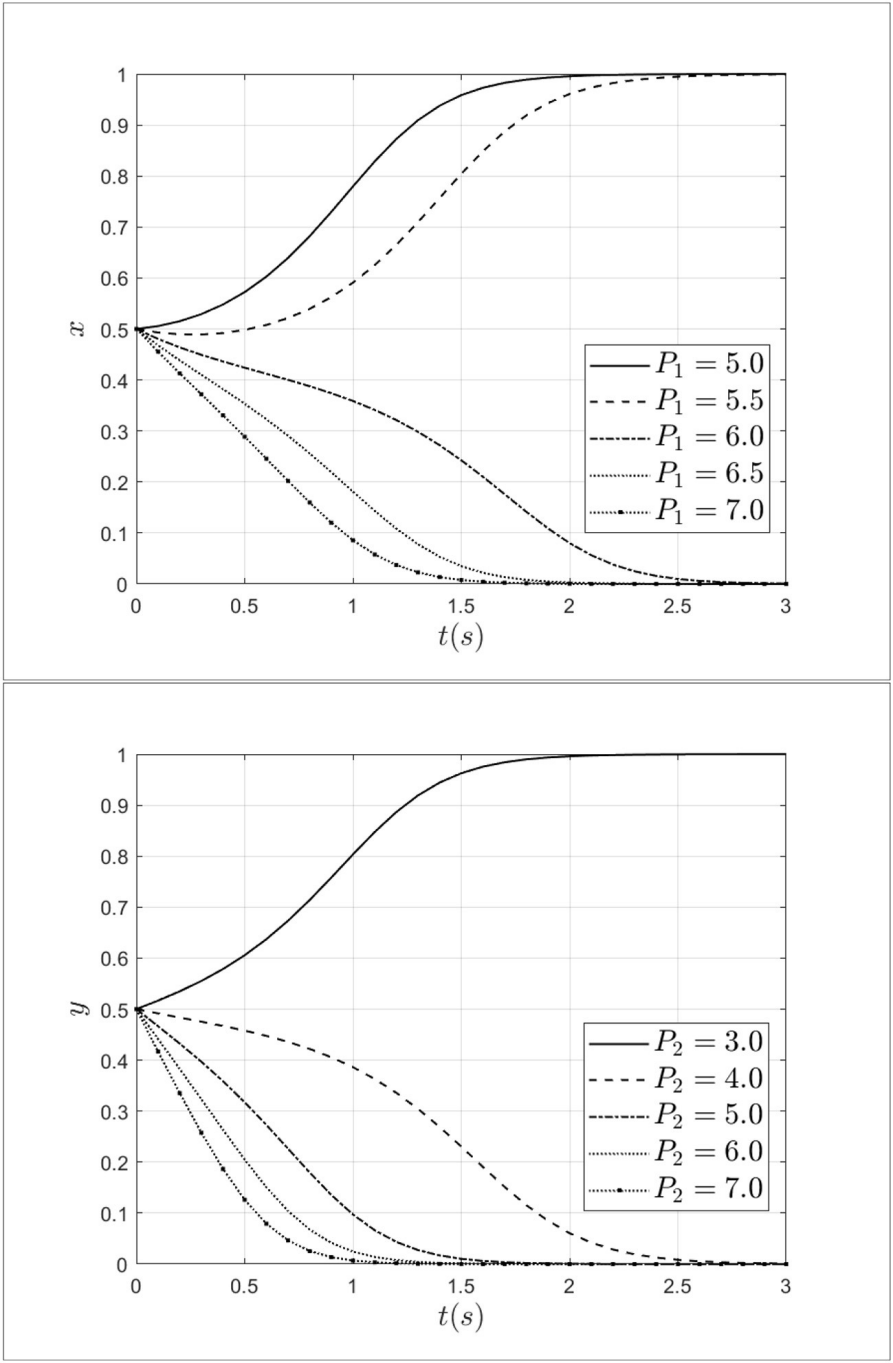
Impact of penalty for patients and doctors on the process of system evolution.

As a regulator, medical insurance institutions not only have the function of paying medical insurance premiums but also have certain regulatory functions. Penalty and reward, as two important measures, have a certain impact on medical fraud behavior between doctors and patients. To explore the impact mechanism of penalty on medical fraud behavior between doctors and patients, the penalty for doctors is taken as 5.0, 5.5, 6.0, 6.5, 7.0 and the penalty for patients is taken as 3.0, 4.0, 5.0, 6.0, 7.0. The impact of penalties for patients and doctors on the process of system evolution is shown in Figure 8.

As shown in Figure 8, when the penalty for doctors increases from 5.0 to 7.0, the doctor's strategy will move from fraud strategy to non-fraud strategy and the rate of moving is gradually accelerating. Similarly, when the penalty for patients increases from 3.0 to 7.0, the patients' strategy will transform from a fraud strategy to a non-fraud strategy. At the same time, as the increase of penalty, the step of the system moving toward a non-fraud state will be shortened. Therefore, it can be seen that penalties for doctors and patients can effectively intervene in the behavior of patients and doctors. Especially, increasing the penalty for doctors and patients can reduce the probability of choosing a fraud strategy.

### 3.4. Impact of reward for patients and doctors

**Figure 9:**
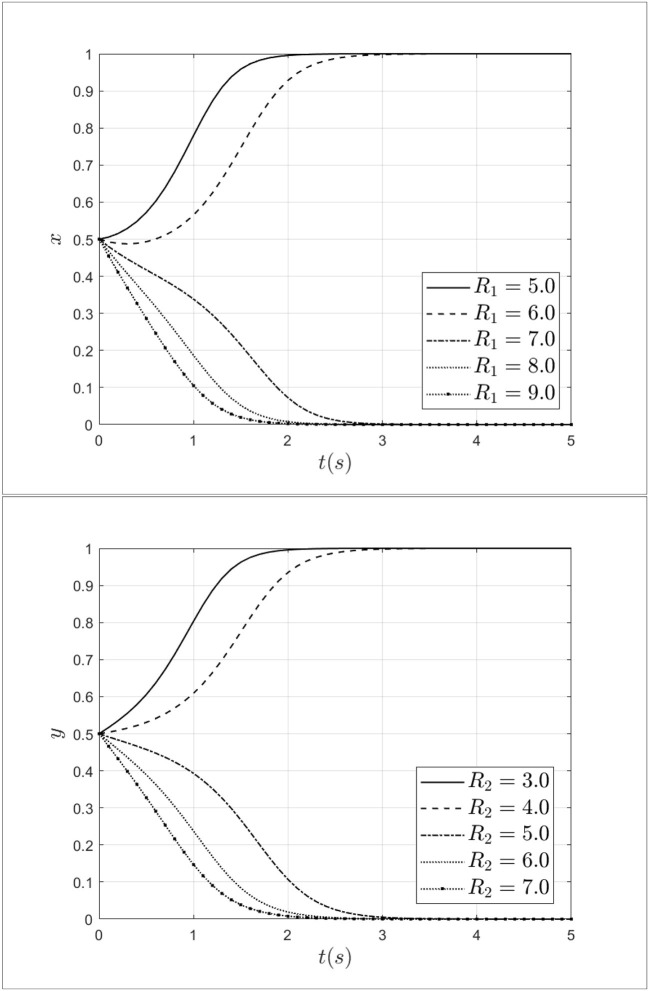
Impact of reward for patients and doctors on the process of system evolution.

To explore the role of reward for patients and doctors during the process of selecting a strategy, this study set the reward for doctors *R*_1_ as 5.0, 6.0, 7.0, 8.0, 9.0 and set the reward for patients *R*_2_ as 3.0, 4.0, 5.0, 6.0, 7,0. The impact of penalties for patients and doctors on the process of system evolution is shown in Figure 9.

As shown in Figure 9, when the reward for doctors *R*_1_ increases from 5.0 to 9.0, doctors' strategy choices will shift from fraud strategy to non-fraud strategy. At the same time, with the increase of *R*_1_ from medical insurance institutions, doctors will gradually opt for a non-fraud strategy. It can be seen that rewards from medical insurance institutions can reduce the probability of choosing a fraud strategy effectively. Besides, when the reward for patients *R*_2_ increases from 3.0 to 7.0, the patients will choose the non-fraud strategy gradually, meanwhile, the rate of choosing the non-fraud strategy will gradually accelerate. Thus, rewards from medical insurance institutions can alleviate and curb medical fraud effectively.

### 3.5. Impact of bribery from patients to doctors

**Figure 10:**
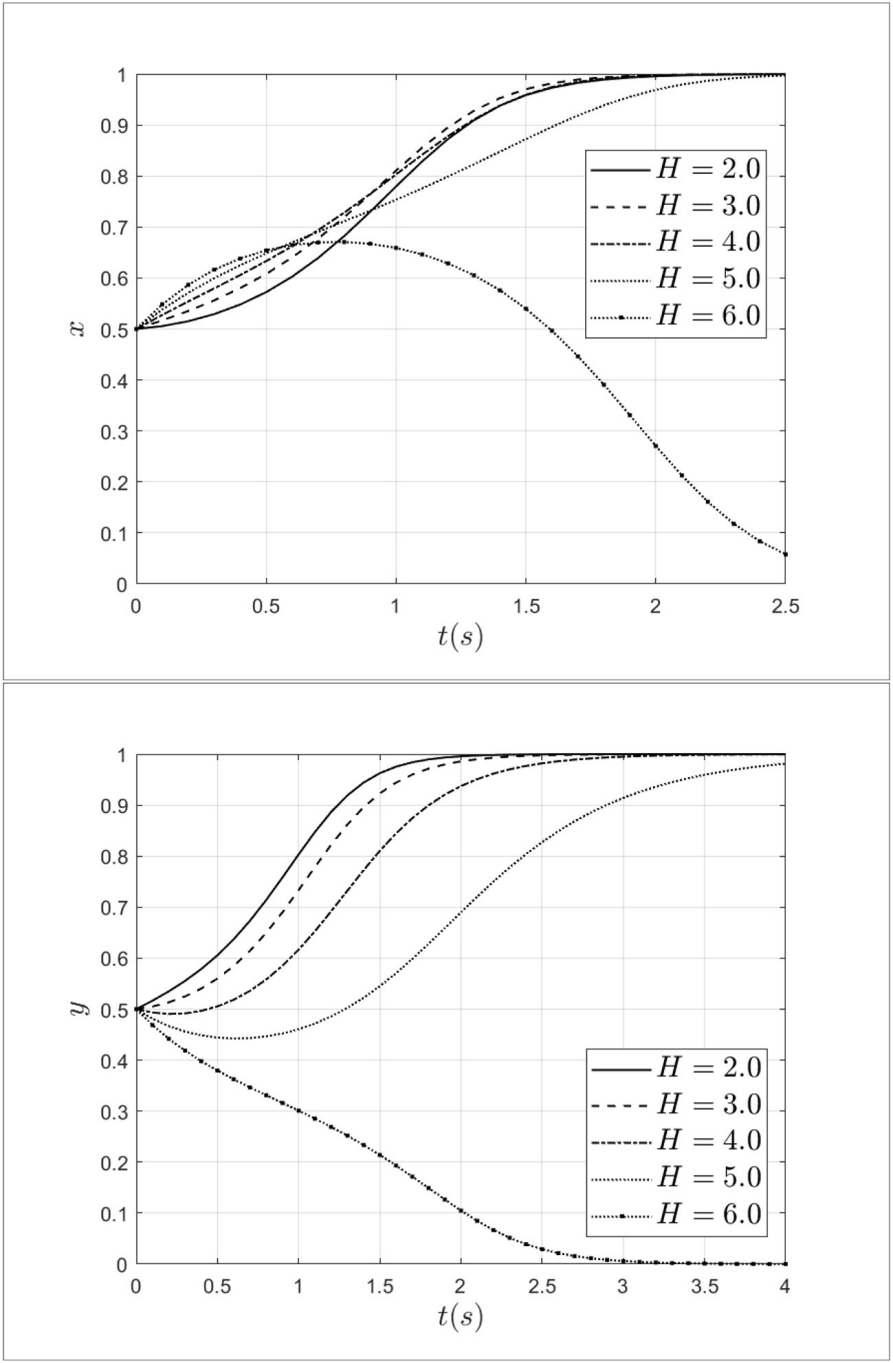
Impact of bribery from patients to doctors on the process of system evolution.

Doctor-patient collusion often cannot be formed without bribes from patients to doctors. To explore the bribery from patients to doctors, this study set bribery as 2.0, 3.0, 4.0, 5.0, and 6.0. Keeping other parameters unchanged, the impact of bribery from patients to doctors on the process of system evolution is shown in Figure 10.

As shown in Figure 10, when the bribery from patients to doctors increases from 2.0 to 3.0, the probability of doctors choosing a fraud strategy will continue to increase. At this moment, doctor-patient collusion is more likely to develop. When bribery increases from 3.0 to 6.0, the rate of doctors choosing a fraud strategy will decrease and the doctors' strategy will transform from a fraud strategy to a non-fraud strategy. In terms of patients, when the bribery from patients to doctors increases from 2.0 to 6.0, the rate of patients choosing a fraud strategy will decrease, and choosing a non-fraud strategy will increase. Especially, when the bribery equal to 6.0, the patients will choose the non-fraud strategy. The above results indicate that the increase of bribery will lead to increase in bribery will lead both doctors and patients to choose a non-fraud strategy.

## 4. Discussion

### 4.1. Conclusion

This study explores the governance of health insurance fraud behavior between doctors and patients using evolutionary game theory. The model is built based on moral hazard from both patients and doctors, penalties and rewards from insurance institutions, bribery from patients, and other factors. The stability of the model is discussed, and the theoretical model is verified through numerical simulations. Additionally, the factors that influence the evolutionary status of the system of health insurance fraud are explored.

The research findings indicate that the evolution of fraudulent behavior in the healthcare system is closely related to the payment matrix initially constructed and the selection of initial parameters for the payment matrix. Increasing the cost of fraudulent behavior for both doctors and patients, reducing moral hazard, increasing penalties for both parties, and increasing rewards can effectively promote a stable strategy for the system to be in a state of non-fraud. In contrast to existing findings (7), this paper finds that reducing moral hazard and increasing the cost of healthcare fraud can also help reduce the incidence of healthcare fraud. Although stakeholders' influence in the policy process is limited (33), this study still aims to provide suggestions for reducing health insurance fraud and building a harmonious healthcare system.

### 4.2. Theoretical findings

This research explored the path of governance in health insurance fraud between patients and doctors. As far as the methodology is concerned, this study was conducted using an evolutionary game approach and assumed finite rationality on the part of both physicians and patients, taking into account the cost of fraud on the part of both physicians and patients, moral hazard, physician-patient collusion, patient bribery, and rewards and penalties on the part of health insurers. The study found that the cost of fraud, moral hazard, penalties, and rewards can effectively influence the evolution of fraud behavior between doctors and patients. Besides, this study provides some methodological and modeling guidance for subsequent research on health insurance fraud, while further broadening the scope and application of evolutionary game theory. Future research can further use mathematical formulas to portray the behavior of health insurance agencies and use three-party games or more cutting-edge game models to conduct more detailed research on health insurance fraud.

### 4.3. Suggestions for government and individual

Based on the conclusions drawn from the analysis, several management inspirations can be implemented to address health insurance fraud. Firstly, it is essential to increase the punishment for patients and doctors who engage in fraudulent behavior. During medical consultations and treatment, doctors and patients may choose to defraud their health insurance for personal gain, which poses a significant challenge to the governance of health insurance and results in a loss of social welfare. Therefore, increasing the penalties for such behavior during medical consultations and treatment can deter doctors and patients from engaging in health insurance fraud and gradually decrease the incidence of fraudulent activity.

Secondly, increasing the fraud costs of doctors and patients. Health care fraud occurs on the one hand because of the low cost of fraud to individuals. Sometimes, people believe that fraud is not easily detected by regulatory agencies, so they will commit medical fraud with impunity, and even more excessively, doctors and patients will engage in medical collusion. Therefore, it is important to increase the cost of doctor-patient fraud. In the event of medical fraud, whether by doctors or patients, they will be put on a credit blacklist. In addition, if a doctor commits medical fraud, his or her professional credentials can be revoked.

Thirdly, reducing moral hazard for patients and doctors is critical to minimize fraudulent behavior. Moral hazard is not only influenced by economic interests but also by the quality of governing officials and the completeness of the supervisory system. To reduce the degree of moral hazard for patients and doctors, it is necessary to promote fraud detection methods and systems actively. Patients and doctors should be guided to establish a working mechanism for non-fraud behavior, which can detect fraudulent behavior early, respond promptly, and resolve it immediately. Additionally, patients and doctors should continually enhance their training and learning to reduce the probability of the moral hazard occurring. Besides, exploring new types of supervision methods, such as the reputation punishment mechanism, is also crucial to effectively reduce the occurrence of fraudulent behavior.

In summary, this article provides theoretical support and practical insights for governance in health insurance fraud. By implementing measures such as increasing punishment for fraudulent behavior, increasing the costs of fraud, and reducing moral hazard for doctors and patients, the incidence of health insurance fraud can be gradually reduced.

### 4.4. Limitations and future directions

Although this study used the evolutionary game theory to explore the behavior of health insurance fraud between doctors and patients. There are still some limitations in the research. Firstly, limited by the limitations of the method, this research is close to reality but does not fully reflect it. In the future, empirical research will be conducted to test the theoretical predictions of the model and validate its assumptions. Secondly, this research explored the behavior of health insurance fraud from a microscopic perspective, future work also can analyze the impact of external factors such as cultural differences, economic development, and political environment on the evolution of health insurance fraud governance. Thirdly, current research doesn't consider the influence of new technology in the governance of health insurance fraud, future work can investigate the impact of technological advancements such as blockchain and AI on the evolution of health insurance fraud governance. Besides, future research also can develop practical tools and guidelines for governments to implement effective governance strategies for health insurance fraud.

## Data availability statement

The raw data supporting the conclusions of this article will be made available by the authors, without undue reservation.

## Author contributions

JL: conceptualization, writing—original draft, and investigation. YW: writing—original draft, investigation, and validation. JY: conceptualization and validation. All authors contributed to the article and approved the submitted version.
